# *Bhlhe40* deficiency attenuates LPS-induced acute lung injury through preventing macrophage pyroptosis

**DOI:** 10.1186/s12931-024-02740-2

**Published:** 2024-02-24

**Authors:** Xingxing Hu, Menglin Zou, Weishuai Zheng, Minghui Zhu, Qinhui Hou, Han Gao, Xin Zhang, Yuan Liu, Zhenshun Cheng

**Affiliations:** 1 Department of Respiratory and Critical Care Medicine, Zhongnan Hospital of Wuhan University, Wuhan, Hubei China; 2 Fourth Ward of Medical Care Center, Hainan General Hospital, Hainan Affiliated Hospital of Hainan Medical University, Haikou, China; 3 Department of Respiratory and Critical Care Medicine, Hainan General Hospital, Hainan Affiliated Hospital of Hainan Medical University, Haikou, China; 4 Wuhan Research Center for Infectious Diseases and Cancer, Chinese Academy of Medical Sciences, Wuhan, Hubei China; 5 Hubei Engineering Center for Infectious Disease Prevention, Control and Treatment, Wuhan, China

**Keywords:** Acute Lung Injury (ALI), Basic helix-loop-helix family member e40 (Bhlhe40), Macrophage, Gasdermin D (GSDMD), Pyroptosis

## Abstract

**Background:**

Acute lung injury (ALI) and its more severe form, acute respiratory distress syndrome (ARDS) as common life-threatening lung diseases with high mortality rates are mostly associated with acute and severe inflammation in lungs. Recently, increasing evidence supports activated inflammation and gasdermin D (GSDMD)-mediated pyroptosis in macrophage are closely associated with ALI. Basic helix-loop-helix family member e40 (Bhlhe40) is a transcription factor that is comprehensively involved in inflammation. However, there is little experimental evidence connecting Bhlhe40 and GSDMD-driven pyroptosis. The study sought to verify the hypothesis that Bhlhe40 is required for GSDMD-mediated pyroptosis in lipopolysaccharide (LPS)-induced inflammatory injury.

**Method:**

We performed studies using *Bhlhe40*-knockout (*Bhlhe40* ^−/−^) mice, small interfering RNA (siRNA) targeting *Bhlhe40* and pyroptosis inhibitor disulfiram to investigate the potential roles of Bhlhe40 on LPS-induced ALI and the underlying mechanisms.

**Results:**

Bhlhe40 was highly expressed in total lung tissues and macrophages of LPS-induced mice. *Bhlhe40*^*−/−*^ mice showed alleviative lung pathological injury and inflammatory response upon LPS stimulation. Meanwhile, we found that *Bhlhe40* deficiency significantly suppressed GSDMD-mediated pyroptosis in macrophage in vivo and in vitro. By further mechanistic analysis, we demonstrated that *Bhlhe40* deficiency inhibited GSDMD-mediated pyroptosis and subsequent ALI by repressing canonical (caspase-1-mediated) and non-canonical (caspase-11-mediated) signaling pathways in vivo and in vitro.

**Conclusion:**

These results indicate Bhlhe40 is required for LPS-induced ALI. *Bhlhe40* deficiency can inhibit GSDMD-mediated pyroptosis and therefore alleviate ALI. Targeting Bhlhe40 may be a potential therapeutic strategy for LPS-induced ALI.

**Supplementary Information:**

The online version contains supplementary material available at 10.1186/s12931-024-02740-2.

## Introduction

Acute lung injury (ALI) is a serious clinical disorder and is characterized by an acute respiratory insufficiency, the symptoms of which include chest pain, chest tightness, and shortness of breath. If the condition is not controlled, it will progress to acute respiratory distress syndrome (ARDS) eventually leading to death, with a mortality rate of greater than 40% [1, 2]. ALI is characterized by an exaggerated host-defense immune response in which influx of inflammatory cells, such as macrophages and neutrophils, into the lung tissue perpetuates a vicious cycle of inflammation that amplifies the accumulation of these cells [3]. Over the years, a great deal of researches has demonstrated that gram-negative bacterial infection is one of the most important factors leading to ALI [4]. Although substantial improvements have been made in comprehending the pathophysiology of ALI over the past decades, effective therapies remain scarce [5]. The reason is that the potential molecular mechanisms driving ALI remain poorly understood. Hence, it is essential to get insight of the molecular mechanisms underlying ALI for further treatment of the condition.

Macrophages are the crucial cell population in mediating inflammation and tissue damage and serve as a key cellular target for the treatment of ALI [6]. During pulmonary homeostasis, the proportion of macrophages in lung immune cells is about 90–95% [7]. In the lung, resident macrophages include alveolar macrophages and interstitial macrophages. Under physiologic conditions, alveolar macrophages comprise the first line of defense in innate immune system against microbes. In ALI, many peripheral macrophages infiltrate into lung tissues and produce various pro-inflammatory cytokines including tumor necrosis factor alpha (TNF-α) and interleukin (IL)-1β [8–10]. Pyroptosis is a highly inflammatory event and the main source of IL-1β [11, 12]. A growing body of evidence suggests that pyroptosis in macrophages may exacerbate the pathophysiological process of ALI [13, 14]. While inhibition of macrophage pyroptosis is thought to efficient for the prevention or treatment of ALI [15, 16].

Pyroptosis is a form of regulated cell death that is both inflammatory and immunogenic and is largely elicited by gasdermin D (GSDMD). Cell pyroptosis protects multicellular organisms from invading pathogenic microbial infections, nevertheless, pyroptosis can cause local and systemic inflammation and even lead to lethal septic shock [17]. When cells are stimulated by inflammatory signals, such as LPS, the downstream inflammasome-associated caspases activated, which cleaves GSDMD. Cleavage of GSDMD leads to the separation of its N-terminal pore-forming domain from the C-terminal repressor domain followed by formation of large pores in the cell plasma membrane, causing cell swelling plasma membrane rupture and facilitating IL-1β release [18, 19]. In most of the cases, GSDMD-mediated pyroptosis is triggered via two upstream pathways, including the caspase-1-mediated inflammasome pathway (i.e., the canonical inflammasome pathway) and the caspase-11-mediated pathway (i.e., the non-canonical pathway) [20]. In the canonical inflammasome pathway, activation of NOD-like receptor family, pyrin domain containing 3 (NLRP3) inflammasome requires both priming and activating steps. Priming is usually mediated by the Toll-like receptor 4 (TLR4)-myeloid differentiation factor 88 (MyD88) pathway, while activating signals promote NLRP3, apoptosis associated speck-like protein containing a CARD (ASC), and pro-caspase-1 to assemble the NLRP3 inflammasome. Upon inflammasome activation, pro-caspase-1 is activated by self-cleavage, and then cleaved caspase-1 mediates cleavage of GSDMD, pro-IL-1β and pro-IL-18. Unlike the classical pathway, in the non-canonical pathway, caspase-11 can be triggered directly by LPS, which can then cleave full-length (GSDMD^FL^) into an N terminal isotype (GSDMD^NT^) [21].

Basic helix-loop-helix family member e40 (Bhlhe40), also known as Dec1, Stra13 and Sharp 2, belongs to the subfamily of transcription factors and is highly conserved across mammalian species. Bhlhe40 binds to target genes at Sp1 element or class B E-box motifs and functions primarily as a transcriptional activator or repressor. An emerging view that Bhlhe40 is an important regulator of inflammation and immunity. Bhlhe40 regulates various immune cellular processes, including macrophage-mediated inflammatory response [22, 23]. In addition, our previous study has reported that *Bhlhe40* deficiency ameliorated pulmonary fibrosis and inflammation through the PI3K/AKT/GSK-3β/β-catenin integrated signaling pathway by using *Bhlhe40* knockout (*Bhlhe40*^*−/−*^) mice [24]. Recently, a study reported that Bhlhe40 deficiency downregulated LPS-induced pyroptosis in periodontal inflammation [25], suggesting a potential relationship between Bhlhe40 and macrophage pyroptosis in ALI.

In the present study, we characterized the expression pattern of Bhlhe40 and investigated for the first time its role in macrophage pyroptosis and ALI. We showed that the expression of Bhlhe40 in LPS-induced lungs and macrophages are significantly increased. *Bhlhe40*^*−/−*^ mice were present decreased macrophages pyroptosis and inflammation and resist to LPS-induced ALI. Mechanistically, *Bhlhe40* deficiency inhibited both caspase-1-mediated and the caspase-11-mediated pathway. These findings revealed that Bhlhe40 plays an essential role in LPS-driven lung inflammation and injury.

## Materials and methods

### Reagents and antibodies

LPS derived from *Escherichia coli* O55:B5 (Cat. No.: HY-D1056) was purchased from MedChem Express (Monmouth Junction, NJ, United States). Recombinant murine M-CSF (Cat. No.:315-02) was obtained from Peprotech (Rocky Hill, NJ, United States). Primers for qRT-PCR were synthesized in Tsingke Biological Technology.Co, Ltd (Beijing, China). Primer details were listed in Quantitative Real-Time PCR (qRT-PCR). Antibodies used for Western blot, immunohistochemistry and Immunofluorescence included Bhlhe40 (NB100-1800SS) from Novus (Centennial, CO, United States), GAPDH (ab181602), GSDMD (ab209845), Caspase-11 (ab180673) from Abcam (Cambridge, United Kingdom), F4/80 (70076T), NLRP3 (15101 S), ASC (67824T) from Cell Signal Technology (Danvers, MA, United States), GSDMD-NT (orb1495171) from biorbyt (Cambridge, United Kingdom), Caspase-1 (A0964) from Abclone (Wuhan, China), and TLR4 (66350-1-Ig), MYD88 (67969-1-Ig) from Proteintech (Rosemont, IL, United States). Anti-rabbit (AS1107) and anti-mouse (AS1106) secondary antibodies conjugated with HRP were from Aspen (Wuhan, China). Sheep anti-mouse/Rabbit IgG polymer (PV-8000) was from ZSGB-BIO (Beijing, China).

### LPS-induced ALI model in mice and drug administration

Wild-type (WT) male C57BL/6J mice aged 6–8 weeks old were purchased from GemPharmatech Co., Ltd (Jiangsu, China). *Bhlhe40* ^*+/−*^ (C57BL/6 background) male and female mice were obtained from Prof. Yang Jian (Nanjing Medical University, Nanjing, China), whose *Bhlhe40*-knockout (*Bhlhe40* ^*−/−*^) mice (RBRC04841) were originally acquired from RIKEN BioResource Center. *Bhlhe40*^ *+/−*^ males and females were mated to obtain *Bhlhe40* ^*+/+*^ and *Bhlhe40*^ *−/−*^ littermates for experiments. All mice used in experiments were male and 6–8 weeks old. All experiments were conducted under pathogen-free conditions on a 12-h light/dark cycle, at a room temperature of 25 ± 2℃ and a relative humidity of 55 ± 5%. All animal experiments in this study were approved by the Institutional Animal Care and Use Committee, Center for Medical Ethics, Wuhan University (Wuhan, China).

LPS-induced ALI model was established by intratracheal LPS administration. Briefly, 40ul LPS solution (5 mg/kg) was delivered into murine trachea after mice were anesthetized with 1% pentobarbital (i.p., 6 ml/kg). The control counterparts were administrated with an identical volume of sterile saline. For disulfiram administration, WT mice were treated with disulfiram at different concentrations or vehicle by intraperitoneal injection on day 1 after intratracheal instillation of LPS.

### Cell isolation and culture

The isolation of bone marrow (BM) cells from *Bhlhe40*^*+/+*^ and *Bhlhe40*^*−/−*^ male mice was performed as described previously [26]. Briefly, BM cells were obtained by flushing mouse femurs and tibias and cultured in RPMI 1640, 10% fetal bovine serum (FBS), 0.05mM β-mercaptoethanol, 1% penicillin/ streptomycin and M-CSF (20 ng/ml) for generation of bone marrow-derived macrophages (BMDMs). Medium was replaced with fresh medium containing M-CSF on day 3 and day 5. BMDMs were harvested on day 7 of culture and incubated for 24 h in medium alone or medium containing LPS (1 μg/ml).

### Knockdown of ***Bhlhe40*** by siRNA transfection

Small interfering RNA (siRNA) targeting *Bhlhe40* and negative control (NC) siRNA were acquired from Ruibo Biotechnology (Guangzhou, China) and were separately mixed with lipo2000 transfection reagent (Invitrogen) according to the manufacturer’s instructions. siRNA-lipo2000 complexes were added to RAW 264.7 cells with Opti-MEM medium. After 8 h, the transfection medium was replaced by culture medium for another 40 h.

### Bronchoalveolar lavage fluid (BALF)

BALF was collected through a tracheal cannula using 0.5 mL PBS for three times per mouse. Samples were centrifuged at 1500 rpm for 7 min at 4◦ C. The supernatant of BALF was stored at -80℃ waiting for testing, and cell pellets were resuspended in PBS counting the total cells using a counter.

### Quantitative real‑time PCR (RT‑qPCR)

Total RNA was extracted from tissues and cells using TRIzol (Invitrogen, Carlsbad, CA, United States) according to the manufacturer’s instructions. RNA reverse transcription was conducted using ReverTra Ace qPCR RT Kit (TOYOBO, Osaka, Japan) and qPCR was performed using UltraSYBR Mixture (CWBIO, Beijing, China). The relative mRNA expression levels were measured on the basis of the Ct value and relative to GAPDH by using the 2^−ΔΔCt^ method.

The mouse primer sequences used in the study were listed as follows: *Bhlhe40*: forward, 5′-CGTTGAAGCACGTGAAAGCA-3′, reverse, 5′-AAGTACCTCACGGGCACAA G-3′; *Il-1β*: forward, 5′-CCGTGGACCT TCCAGGATGA-3′, reverse, 5′-GGGAACGTCACACACCAGCA-3′; *Il-6*: forward, 5′-AGTTGCCTTCTTGGGACTGA-3′, reverse, 5′-TCCACG ATTTCCCAGAGAAC-3′; *Tnf-α*: forward, 5′-CATCTTCTCAAAATTCG AGTGACAA-3′, reverse, 5′-TGGGAGTAGACAAGGTACAACCC-3′; *Ifn-γ*: forward, 5′-GCCACGGCACAGTCATTGA-3′, reverse, 5′-TGCTG ATGGCCTGA TTGTCTT-3′; *Mcp-1*: forward, 5′-TACAAGAGGATCAC CAGCAGC-3′, reverse, 5′-ACCTTAGGGCAGATGCAGTT-3′; *Cxcl10*: forward, 5′-ATGACGGGCCAGTGAG AATG-3′, reverse, 5′- CGGATTC AGACATCTCTGCTCAT-3′; *Il-10*: forward, 5′- CTTACTGACTGGCAT GAGGATCA-3′, reverse, 5′- GCAGCTCTAGGAGCATGTGG-3′; *Gsdmd*: forward, 5′- TTCCAGTGCCTCCATGAATGT-3′, reverse, 5′- GC TGTGGACCTCAGTGATCT-3′; *Casp1*: forward, 5′-CAAGGCACGGG ACCTATGTG-3′, reverse, 5′- TCCTGCCAGGTAGCAGTCTT-3′; *Casp11*: forward, 5′-AGCGTTGGGTTTTTGTAGATGC-3′, reverse, 5′-CCTTGTGAACTCTTCAGGGGA-3′; *Nlrp3*: forward, 5′-ATCAACAG GCGAGACCTCTG-3′, reverse, 5′-GTCCTCCTGGCATAC CATAGA-3′; *Asc*: forward, 5′-GCTACTATCTGGAGTCGTATGGC-3′, reverse, 5′-GA CCCTGGCAATGAGTGCTT-3′; *iNOS*: forward, 5′- TCCCTTCCGAAG TTTCTG GC-3′, reverse, 5′-ACGTAGACCTTGGGTTTGCC-3′; *Gapdh*: forward, 5′-TGAAGG GTGGAGCCAAAAG-3′, reverse, 5′-AGTCTTCT GGGTGGCAGTGAT-3′.

### Enzyme-linked immunosorbent assay (ELISA)

The protein levels of IL-1β and IL-10 in BALF and supernatant were detected by enzyme-linked immunosorbent assay (ELISA) kit according to the instructions of the kits (BlueGene Biotech, Shanghai, China).

### Western blot

Proteins were extracted from lung tissues and BMDMs using RIPA lysis buffer (Sigma-Aldrich, St. Louis, MI, United States) supplemented with PMSF (Beyotime, Shanghai, China). Tissue and cell lysates were centrifuged at 12,000×g for 15 min at 4℃, and then the supernatants were collected. For Western blot assay, proteins were subjected to SDS-PAGE, transferred onto PVDF membranes (Millipore, Bedford, MA, United States), blocked with 5% skim milk for 1 h at room temperature, and subsequently incubated with the indicated primary antibodies overnight at 4℃. The membranes were then incubated with HRP-conjugated anti-rabbit and anti-mouse secondary antibodies for 1 h at room temperature. Subsequently, the bands were visualized with an enhanced ECL kit (Thermo Fisher Scientific, Waltham, MA, United States), and then exposed to the electrochemiluminescence (ECL) system (Tanon, Shanghai, China).

### Histopathology and immunohistochemistry analysis

The left lungs were fixed with 4% formaldehyde, followed by alcohol gradient dehydration and dewaxing, paraffin-embedded sections with a thickness of 4 μm were prepared. Then, hematoxylin and eosin (H&E) were performed using standard techniques and the pathological changes of lung tissues were observed under light microscope. The degree of lung injury was graded on a scale of 0–4 (0, absent and appears normal; 1, light; 2, moderate; 3, strong; 4, intense) for interstitial edema and neutrophil infiltration. Moreover, a total lung injury score was calculated as the sum of the two components (three sections from each lung), as described previously [27]. Evaluations were performed by two pathologists blind to experimental groups. For immunohistochemistry (IHC), lung sections were stained with anti-Bhlhe40 antibody at 4℃ overnight, then washed and incubated with secondary antibody (Sheep anti-mouse/Rabbit IgG polymer) for 1 h at room temperature. Positive stained area was blindly determined by two pathologists.

### Immunofluorescence

For tissue immunofluorescent double staining, formalin-fixed and paraffin-embedded sections were deparaffinized in xylene, hydrated with an ethanol gradient and briefly washed with distilled water. Paraffin sections were placed in a repair box filled with EDTA antigen retrieval buffer (pH 8.0) and heated in a microwave oven for antigen retrieval. Next, the sections were incubated with goat serum for 30 min, followed by incubation with primary antibodies for Bhlhe40, GSDMD-NT and F4/80 at 4℃ overnight. The next day, the sections were incubated with Goat Anti-Rabbit IgG H&L(HRP) for 50 min at room temperature in the dark, then TSA staining using iFluor® 647 tyramide or iFluor® 488 tyramide, and nuclear staining with DAPI was performed for 10 min. Finally, images of immunofluorescence (IF) staining were taken using a fluorescence microscope (Olympus BX53).

### Statistical analysis

Data statistical analysis was performed using Prism 8.0 software. Values are expressed as mean ± SEM. Comparisons between 2 groups were performed by the two-tailed Student’s t test. Multiple group comparisons with one variable and two variables were performed by one-way ANOVA and two-way ANOVA followed by Bonferroni’s multiple comparisons test, respectively.

## Results

### Bhlhe40 is upregulated in lung tissues and macrophages in LPS-induced ALI

**Fig. 1:**
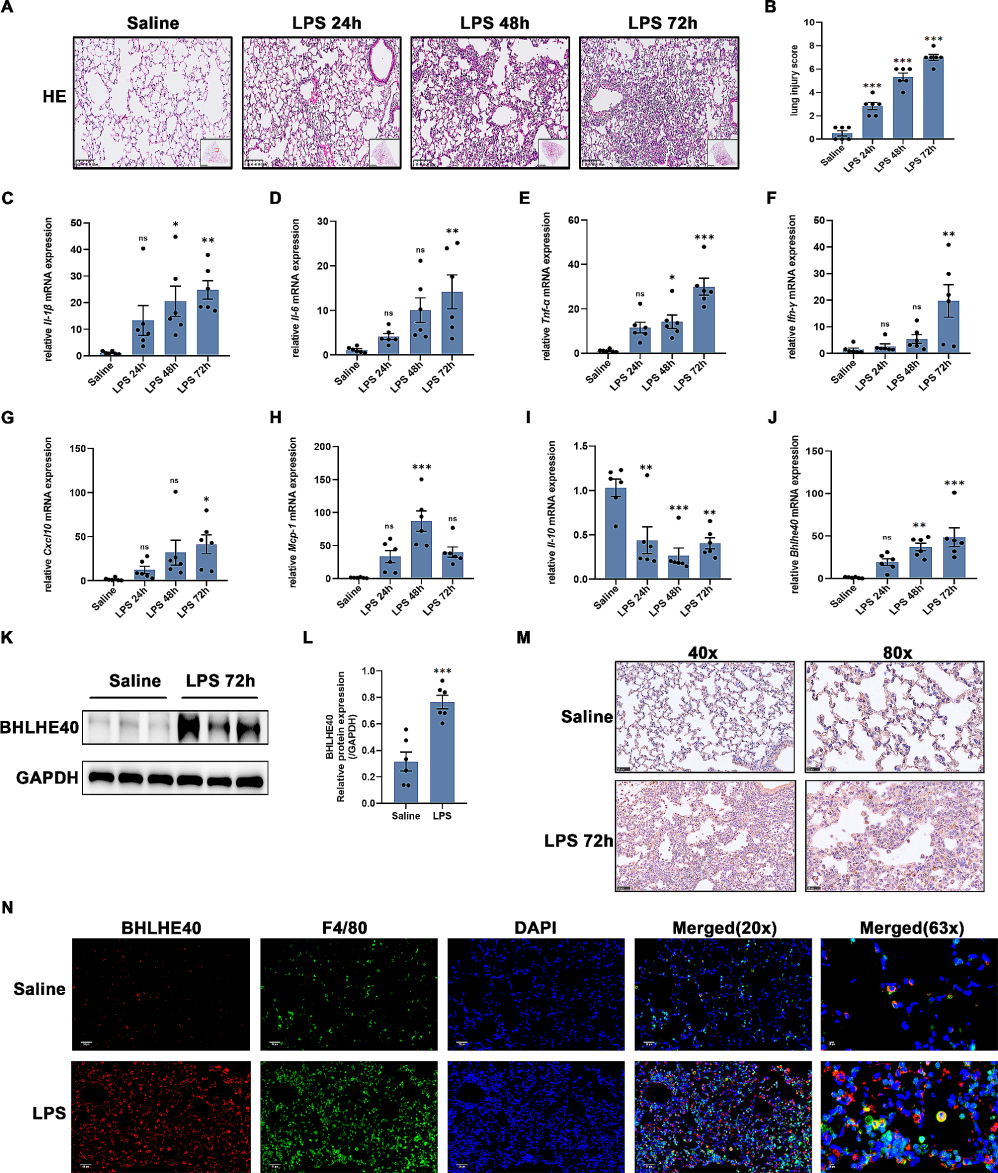
Bhlhe40 is upregulated in lung tissues and macrophages in LPS-induced ALI. (**A**) Representative Hematoxylin-eosin staining (H&E) of lung tissues in mice stimulated by LPS for different times. Scale bar = 100 μm. (**B**) Lung injury score based on H&E staining. (**C**-**I**) Relative mRNA levels of inflammatory cytokines (*Il-1β, Il-6, Tnf-α, Ifn-γ*, *Cxcl10, Mcp-1, Il-10*) in the lungs of mice after LPS induction for different times. (**J**-**L**) qRT-PCR and Western blots and statistical analyses of Bhlhe40 in the lungs of mice after LPS induction for different times. (**M**) Representative images of immunohistochemistry for Bhlhe40 expression in the lungs of mice. Scale bar = 50 μm and 25 μm. (**N**) Representative images of F4/80 and Bhlhe40 double-immunofluorescence staining of LPS-induced lungs. Scale bar = 50 μm and 20 μm. *n* = 6. Data are shown as the mean ± SEM. Statistical analysis was performed by one-way ANOVA followed by Bonferroni’s multiple comparisons test or unpaired two-tailed Student’s t-test. *p < 0.05, **p < 0.01, ***p < 0.001, ^*ns*^*p* > 0.05

In the present study, we firstly investigated the effects of LPS intervention on lung histopathologic characters in mice at different times. The LPS-challenged mice appeared increased septal thickness, intra-alveolar transudates, and increased inflammatory cell infiltration in a time-dependent manner within 72 h after LPS induction (Fig. 1A). Compared with the control group, the lung injury score after LPS stimulation increased gradually over time (Fig. 1B). To further evaluate the degree of lung tissue inflammation, we detected the expression of a series of inflammatory cytokines in lung tissue at specified times after LPS administration. The mRNA expressions of pro-inflammatory cytokines *Il-1β, Il-6, Tnf-α, Ifn-γ* and *Cxcl10* were significantly increased in a time-dependent manner within 72 h post LPS induction (Fig. 1C-G), while *Mcp-1* mRNA expression peaked at 48 h post LPS intervention (Fig. 1H). Additionally, anti-inflammatory cytokines *Il-10* mRNA expression decreased to the lowest at 48 h post LPS stimulation, and slightly increased at 72 h (Fig. 1I). The above results indicated that the degree of lung tissue inflammation exacerbated in a time-dependent manner after LPS administration.

To explore the possible involvement of Bhlhe40 in ALI, we detected the level of Bhlhe40 in lung tissues from LPS-treated mice. Both the mRNA and protein expression levels of Bhlhe40 were significantly upregulated in lungs of mice treated with LPS (Fig. 1J-L). These changes were synchronized with the inflammatory levels, including IL-1β and TNF-α, suggesting that Bhlhe40 may act as a regulator of inflammation response in ALI. Moreover, Bhlhe40 localization and expression were further confirmed by immunohistochemistry and immunofluorescence analysis using F4/80 as a macrophage marker. We found that the increased Bhlhe40 was mainly localized in macrophages (Fig. 1M-N). Collectively, these results suggest that the expression of Bhlhe40 is markedly augmented in LPS-induced ALI in mice.

### *Bhlhe40* deficiency increases resistance to LPS-induced ALI in mice

**Fig. 2:**
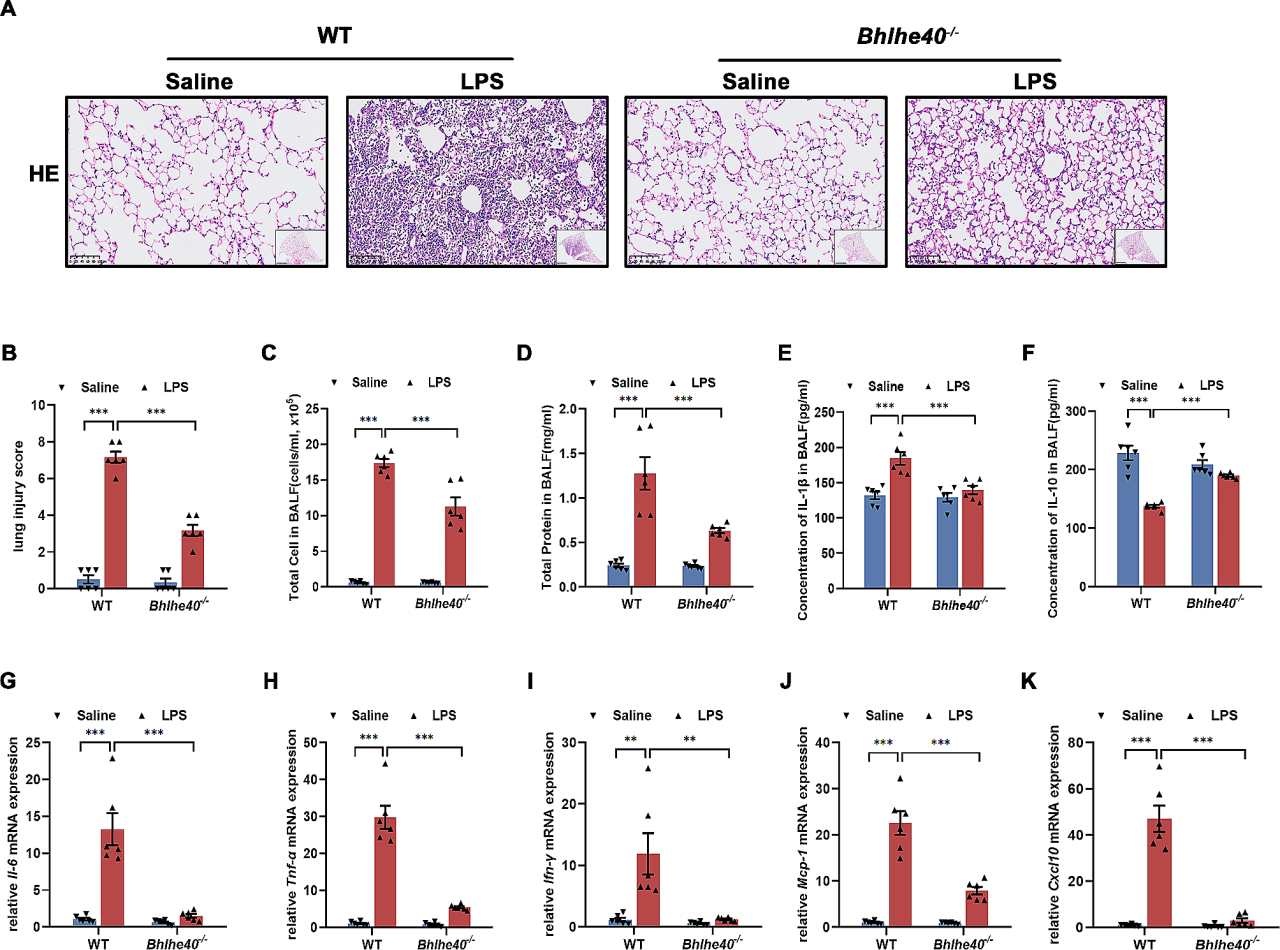
*Bhlhe40* deficiency increases resistance to LPS-induced ALI in mice. (**A**) Representative Hematoxylin-eosin staining (H&E) of lung tissues in mice after LPS induction. Scale bar = 100 μm. (**B**) Lung injury score based on H&E staining. (**C**) Total number of cells counted in the BALF of mice. (**D**) Total protein concentration in the BALF of mice. (**E**) IL-1β levels in BALF of mice. (**F**) IL-10 levels in BALF of mice. (**G**-**K**) Relative mRNA levels of inflammatory cytokines (*Il-6, Tnf-α, Ifn-γ*, *Mcp-1, Cxcl10*) in the lungs of mice after LPS induction. *n* = 6. Data are shown as the mean ± SEM. Statistical analysis was performed by two-way ANOVA followed by Bonferroni’s multiple comparisons test. **p < 0.01, ***p < 0.001

To investigate the role of Bhlhe40 in a murine model of acute lung injury, wild type (WT) and *Bhlhe40*^*−/−*^ mice were employed and randomized to LPS or saline. After 72 h of LPS induction, lung tissues were collected for HE staining and then evaluated the lung injury scores (Fig. 2A-B). The results suggested that the alveolar structures in Saline-treated WT and *Bhlhe40*^*−/−*^ mice were normal, while LPS-stimulated WT mice showed a disordered alveolar structure in lungs characterized by obvious edema, alveolar septal thickening, as well as inflammatory cell infiltration, which were significantly abrogated in LPS-stimulated *Bhlhe40*^*−/−*^ mice. In addition, WT mice exhibited higher levels of total cells and total protein in BALF than *Bhlhe40*^*−/−*^ mice followed by LPS treatment (Fig. 2C-D). Bhlhe40 has previously been found to regulate secreted factors such as cytokines or chemokines [28]. Therefore, we examined the mRNA expression of cytokines and chemokines in lung tissues of the different groups, such as *Il-6, Tnf-α, Ifn-γ, Mcp-1, Cxcl10* and detected the secretion of IL-1β and IL-10 in BALF by ELISA. *Bhlhe40*^*−/−*^ mice showed markedly lower expressions of *Il-6, Tnf-α, Ifn-γ, Mcp-1* and *Cxcl10* than WT mice followed by LPS induction (Fig. 2G-K**).** Notably, compared with WT mice, *Bhlhe40*^*−/−*^ mice showed higher levels of IL-10 release but lower levels of IL-1β in BALF after LPS stimulation (Fig. 2E-F). Thus, these results demonstrate that *Bhlhe40* deficiency ameliorates LPS-induced lung injury and cytokines production.

### *Bhlhe40* deficiency alleviates GSDMD-mediated pyroptosis of the lung in LPS-induced ALI mice

The downregulation of IL-1β release indicated the involvement of pyroptosis [29]. To investigated whether Bhlhe40 modulates pyroptosis in LPS-induced ALI, we first detected the expression of GSDMD and other gasdermin family members, including GSDMA, GSDMC and GSDME. LPS increased *Gsdmd* mRNA expression without affecting *Gsdmc* and *Gsdme* in mice lungs. In contrast, *Gsdma* expression was downregulated after LPS stimulation (Supplementary Fig.S1A), suggesting the potential role of GSDMD-mediated pyroptosis in LPS-induced ALI. Disulfiram is a selective inhibitor for GSDMD-mediated pyroptosis by blocking pore formation [15]. As expect, disulfiram was sufficient to inhibited LPS-induced lung inflammations in a dose-dependent manner by detecting various pro-inflammatory cytokines, including *Il-6, Tnf-α, Mcp-1* and *Cxcl10* (Supplementary Fig.S1B). This is similar to the earlier observation in a model of ALI induced by intraperitoneal injection of LPS by Jiping Zhang et al. [30].

**Fig. 3:**
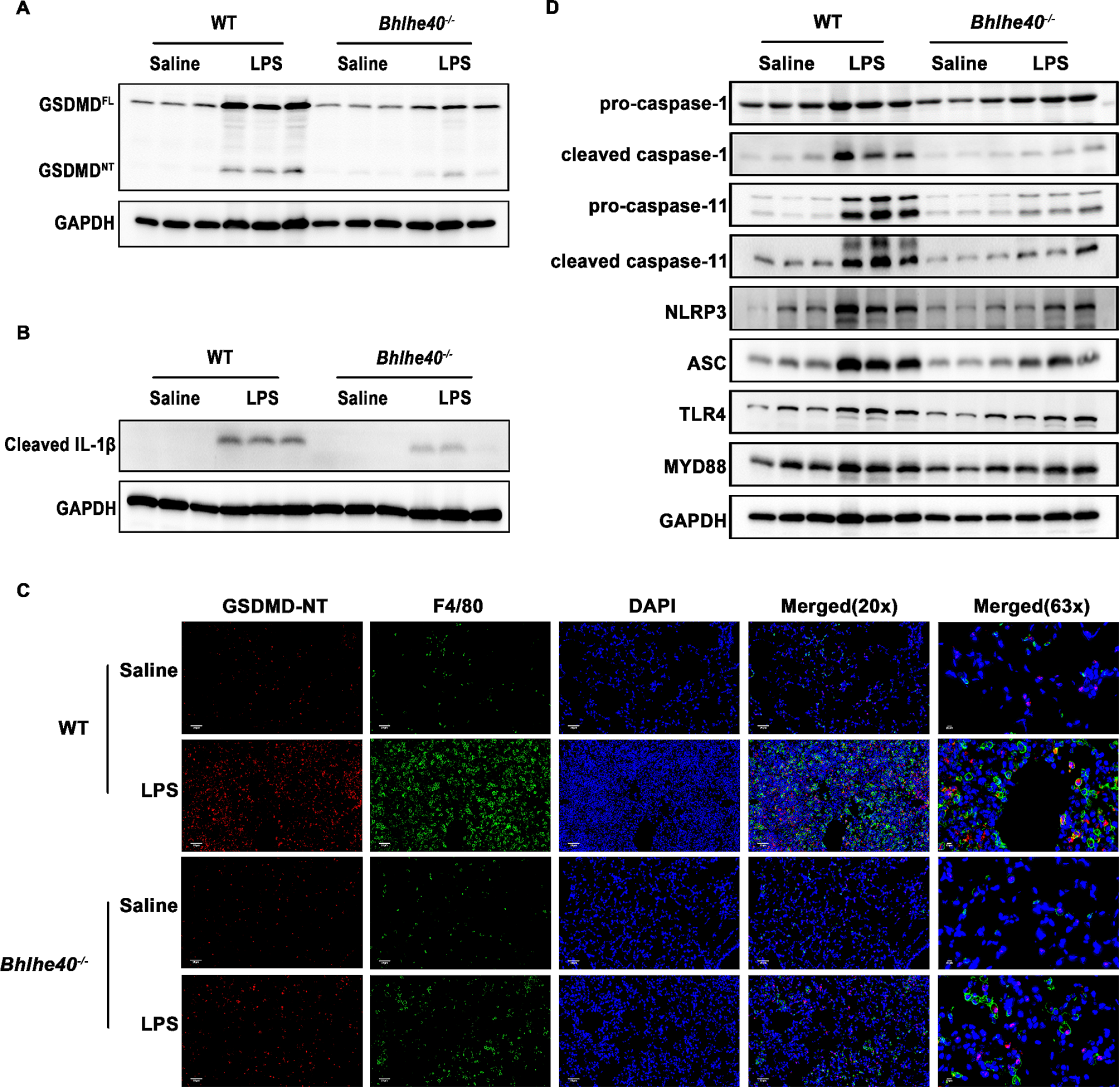
*Bhlhe40* deficiency alleviates GSDMD-mediated pyroptosis and caspase-1 and caspase-11 pathways in LPS-induced ALI mice. (**A**) Representative Western blot of GSDMD^FL^ and GSDMD^NT^ in the lung tissues of mice. (**B**) Representative Western blot of cleaved IL-1β in the lung tissues of mice. (**C**) Representative images of F4/80 and GSDMD^NT^ double-immunofluorescence staining of LPS-induced lungs. Scale bar = 50 μm and 20 μm. (D) Representative Western blot of pro-caspase-1, cleaved caspase-1, pro-caspase-11, cleaved caspase-11, NLRP3, ASC, TLR4 and MYD88 in the lung tissues of mice. *n* = 6

We next investigated whether the mitigation of lung injury in *Bhlhe40*^*−/−*^ mice was associated with pyroptosis by immunoblot analysis of GSDMD protein expression and its activated cleaved fraction (GSDMD^NT^). After LPS stimulation, we observed an increased expression of GSDMD^FL^ and a detected band of GSDMD^NT^ in mice, suggesting pyroptosis occurred during LPS-induced ALI (Fig. 3A and Fig.S2A). Compared with WT mice, *Bhlhe40* depletion significantly reduced the expression of GSDMD^NT^, as well as GSDMD^FL^ (Fig. 3A **and Fig.S2A).** In addition, *Bhlhe40*^*−/−*^ mice exhibited lower expression of cleaved IL-1β in lung tissues (Fig. 3B and Fig.S2B). To test whether GSDMD-mediated pyroptosis occurred in lung macrophages, we performed immunofluorescence with an antibody specific for GSDMD^NT^ and detected it in some macrophages (marked by F4/80) after LPS induction. Compared with WT mice, *Bhlhe40*^*−/−*^ mice exhibited decreased numbers of macrophages expressed GSDMD^NT^ (Fig. 3C**).** These data suggested that *Bhlhe40* deficiency inhibited GSDMD-mediated pyroptosis in macrophage during ALI.

### *Bhlhe40* deficiency inhibits GSDMD-mediated pyroptosis through both canonical and non-canonical pathways in LPS-induced ALI mice

Previous studies demonstrated that GSDMD-mediated pyroptosis was induced by LPS through caspase-1 dependent canonical pathway and the caspase-11 mediated pathway [31]. In the present study, we first examined indispensable components of the caspase-1-mediated inflammasome pathway in lung tissues of the different groups, depicting significant increase in the protein levels of pro-caspase-1, cleaved caspase-1, NLRP3, ASC, TLR4 and MYD88 in the LPS-stimulated WT mice compared with the Saline-stimulated WT mice. However, the dramatic upregulation of cleaved caspase-1, NLRP3 and ASC expression levels was obviously blunted in LPS-stimulated *Bhlhe40*^*−/−*^ mice, whereas TLR4 and MYD88 levels had no significant alteration (Fig. 3D and Fig.S2C). Notably, the mRNA levels of *Casp1*, *Nlrp3* and *Asc* were regulated consistently with the corresponding protein results (Fig.S2D), suggesting Bhlhe40 is required for up-regulating transcriptions of NLRP3 inflammasome components.

We next determined the effects of *Bhlhe40* deficiency on caspase-11-mediated pathway in the lung. LPS upregulated *Casp11* mRNA expression in the lungs of WT mice, but this LPS-induced upregulation was inhibited in lungs of *Bhlhe40*^*−/−*^mice (Fig.S2D). At the protein levels, LPS significantly upregulated pro-caspase-11and cleaved caspase-11, while the expressions induced by LPS were markedly abrogated in *Bhlhe40*^*−/−*^ mice (Fig. 3D and Fig.S2E). Taken together, our findings indicate that *Bhlhe40* deficiency suppresses GSDMD-mediated pyroptosis through both canonical and non-canonical pathways.

### *Bhlhe40* deficiency inhibits GSDMD-mediated pyroptosis through both canonical and non-canonical pathways in macrophages

**Fig. 4:**
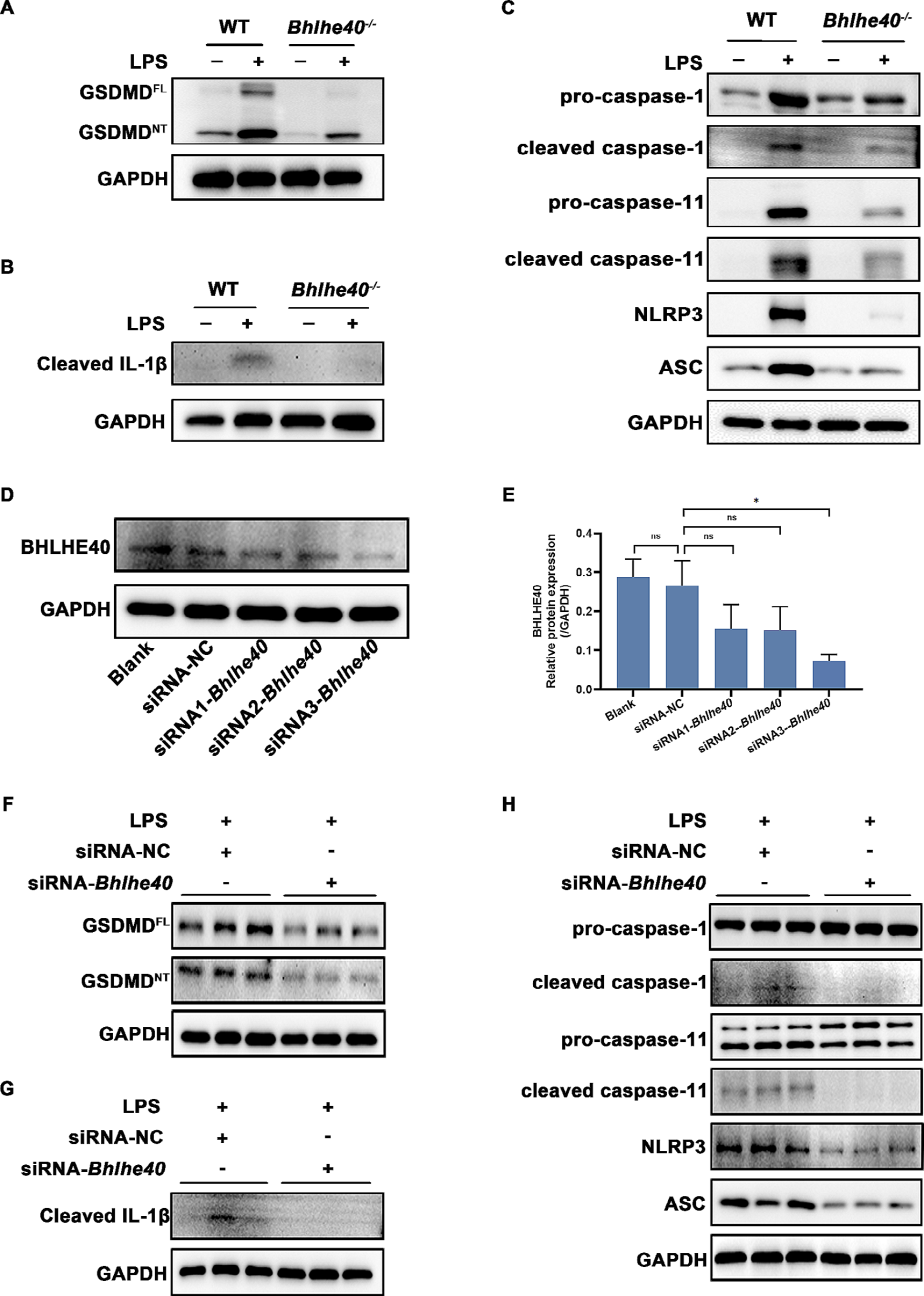
*Bhlhe40* deficiency inhibits GSDMD-mediated pyroptosis through both canonical and non-canonical pathways in BMDMs and in RAW264.7 cell line. (**A**-**C**) Representative Western blot of GSDMD^FL^, GSDMD^NT^, cleaved IL-1β, pro-caspase-1, cleaved caspase-1, pro-caspase-11, cleaved caspase-11, NLRP3 and ASC in BMDMs. (**D**-**E**) RAW264.7 cells were transfected with three *Bhlhe40* siRNAs (siRNA#1, siRNA#2 and siRNA#3) or normal control siRNA (NC) for 48 h. The protein levels of Bhlhe40 were detected by western blots and quantified analysis. (**E**-**F**) Representative Western blot of GSDMD^FL^, GSDMD^NT^, cleaved IL-1β, pro-caspase-1, cleaved caspase-1, pro-caspase-11, cleaved caspase-11, NLRP3 and ASC in RAW246.7 cells. *n* = 3. Data are shown as the mean ± SEM. Statistical analysis was performed by one-way ANOVA followed by Bonferroni’s multiple comparisons test. *p < 0.05, ^*ns*^*p* > 0.05

To further investigated the role of Bhlhe40 in GSDMD-mediated pyroptosis in macrophage, bone marrow cells were derived from WT and *Bhlhe40*^*−/−*^ mice for generated BMDMs with M-CSF. We first analyzed the Bhlhe40 expression in BMDMs with or without stimulation by LPS. Bhlhe40 was highly expressed in LPS-treated BMDMs (Fig.S3A-B). Consistently with our in vivo results, *Bhlhe40* knockout significantly decreased the protein levels of GSDMD^NT^ and cleaved IL-1β after LPS stimulation in BMDMs (Fig. 4A-B **and Fig.S3C-D)**. *Bhlhe40* deficiency also inhibited the canonical and non-canonical pathways by decreased the expression of pro-caspase-1, cleaved caspase-1, pro-caspase-11, cleaved caspase-11, NLRP3 and ASC (Fig. 4C **and Fig.S3E)**.

To avoid the potential development impact of *Bhlhe40* knockout on BMDMs [32] and further verify the reliability of previous results, transfection siRNA targeting *Bhlhe40* was performed in RAW 264.7 cell line. Compared with normal control (NC), siRNA#3 reduced Bhlhe40 protein level by 80% (Fig. 4D-E). Therefore, siRNA#3 was chosen for following experiments. *Bhlhe40* knockdown in RAW 264.7 cells significantly decreased the protein levels of GSDMD^NT^ and cleaved IL-1β upon LPS stimulation (Fig. 4F-G). Additionally, the protein levels of cleaved caspase-1, cleaved caspase-11, NLRP3 and ASC were also dramatically downregulated by *Bhlhe40* knockdown (Fig. 4H), suggesting that *Bhlhe40* deficiency repressed GSDMD-mediated pyroptosis through both canonical and non-canonical pathways in macrophages.

### ***Bhlhe40*** deficiency suppresses LPS-induced inflammatory cytokine production in macrophages

**Fig. 5:**
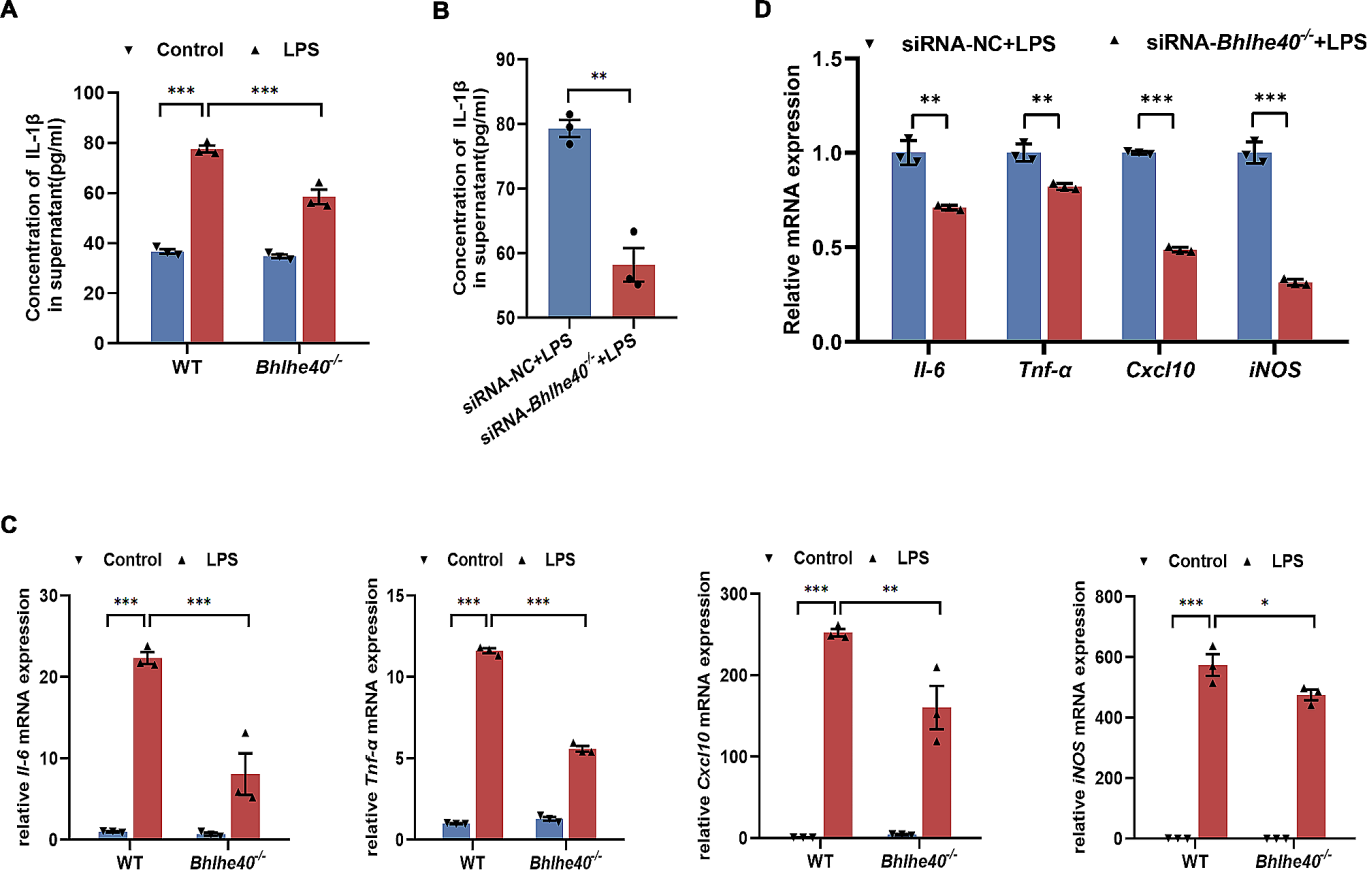
*Bhlhe40* deficiency suppresses LPS-induced inflammatory cytokine production in BMDMs and in RAW264.7 cell line. (**A**) Il-1β levels in the culture supernatant of BMDMs. (**B**) Il-1β levels in the culture supernatant of RAW264.7 cells. (**C**) Relative mRNA levels of *Il-6, Tnf-α, Cxcl10 and iNOS* in BMDMs were assessed by qRT-PCR. (**D**) Relative mRNA levels of *Il-6, Tnf-α, Cxcl10 and iNOS* in RAW264.7 cells were assessed by qRT-PCR. *n* = 3. Data are shown as the mean ± SEM. Statistical analysis was performed by two-way ANOVA followed by Bonferroni’s multiple comparisons test or unpaired two-tailed Student’s t-test. *p < 0.05, **p < 0.01, ***p < 0.001

We next examined the content of IL-1β in the culture supernatant of BMDMs treated with LPS by ELISA. Compared with WT group, *Bhlhe40* deficiency sufficiently inhibited the release of IL-1β from BMDMs (Fig. 5A). Similar results were obtained in RAW264.7 culture supernatant when knockdown *Bhlhe40* was performed using siRNA (Fig. 5B). Additionally, *Bhlhe40*^*−/−*^ BMDMs showed lower mRNA expression of pro-inflammatory mediators, including *Il-6, Tnf-α, Cxcl10 and iNOS*, than WT BMDMs following LPS challenge (Fig. 5C). Similar results were obtained in RAW264.7 cells, which showed lower mRNA levels of *Il-6, Tnf-α, Cxcl10 and iNOS* in *Bhlhe40*-knockdown group than in NC group after LPS incubation (Fig. 5D). These data suggested that *Bhlhe40* deficiency attenuated LPS-induced inflammation damage in macrophages.

## Discussion

In this study, we unveiled that Bhlhe40 is upregulated in mice following LPS-induced ALI. Specifically, Bhlhe40 is mainly overexpressed in macrophages in inflammatory lung tissues. And *Bhlhe40* deficiency significantly ameliorates tissue damage and suppresses macrophage pyroptosis in LPS-induced ALI in mice. In line with the in vivo data, *Bhlhe40* knockout exerts similar protective effects on LPS-induced inflammatory response and pyroptosis in macrophage. In terms of mechanism, we identify that *Bhlhe40* deficiency inhibits GSDMD-mediated pyroptosis through both caspase-1-mediated inflammasome pathway and the caspase-11-mediated pathway. Be based upon these results, we suppose that Bhlhe40 may be a promising therapeutic target for the prevention of LPS-induced ALI.

The inflammatory response is a crucial component in the pathogenesis of ALI, its pathophysiological features are inflammatory exudation and an imbalance between pro-inflammatory and anti-inflammatory responses [33]. Previous studies showed that Bhlhe40 is involved in the development of inflammatory diseases such as periodontal inflammation, myocardial inflammation, and rheumatoid arthritis [34–36]. Macrophages form an essential component of the innate immune system, and have been reported to govern the fate of organs during inflammation and injury, including the lung [37, 38]. Recent studies identified Bhlhe40 as an important transcriptional regulator in macrophage. For example, Jarjour et al. reported that *Bhlhe40*-deficient mice have a mildly reduced peritoneal macrophage compartment [39]. However, other study reported that *Bhlhe40*^*−/−*^ alveolar macrophages were able to maintain their numbers in a steady-state but showed evidence of a proliferative defect in the present of competition with WT cells [32]. This may reflect the complex role of Bhlhe40 in macrophages regulation. In the present study, we demonstrated that Bhlhe40 was required for lung macrophage pyroptosis, further supported Bhlhe40 as an important and unique regulator in macrophage regulation.

Accumulating evidence demonstrates that inflammation and cell death interplay with each other to create a cycle of auto-amplification that leads to further expansion of the inflammatory response [40]. Pyroptosis, a recently identified type of programmed cell death, is triggered by pro-inflammatory signals and associated with inflammation. In the light of previous studies, GSDMD is the executioner of pyroptosis, and GSDMD cleavage is the key step of pyroptosis [41, 42]. Pyroptosis occurs in both lung parenchymal cells and immune cells, but the latter are established as critical executors [38, 43]. Extensive studies have indicated macrophage pyroptosis plays a crucial role in the pathogenesis of ALI [44–46], furthermore, GSDMD deletion has consistently alleviated inflammation-driven diseases in various mouse models, including ALI [30, 47]. One recent study has reported that *Bhlhe40* deficiency impaired the LPS-induced expression of IL-1β and the activation of GSDMD in periodontal ligament fibroblasts [25]. The aforesaid series of researches has suggested that Bhlhe40 may play a role in macrophage pyroptosis and, thus, participant in ALI. Consistent with above findings, we found that GSDMD-dependent macrophage pyroptosis occurred in the LPS-induced ALI model, whereas it was inhibited when *Bhlhe40* was knocked out in mice. Moreover, the above in vivo findings were further verified by in vitro observations in BMDMs and macrophage cell lines. Collectively, these findings indicate that Bhlhe40 is a key regulator GSDMD-mediated macrophage pyroptosis of in ALI. Notably, *Bhlhe40*^*−/−*^ mice and macrophages exhibited downregulation of some non-pyroptosis inflammatory factors. Although pyroptosis inhibitor also inhibited these inflammatory factors in our findings, we cannot exclude other regulatory pathways are involved.

Pyroptosis pathways mainly includes canonical and non-canonical pathways. The canonical pathway is intrinsically dependent on caspase-1 activation and mediated by the NLRP3 inflammasome. In the non-canonical pathway, caspase-11 can specifically bind to LPS, leading to pyroptosis [48]. It is worth noting that GSDMD is the common downstream executor of the canonical and non-canonical pathways [18]. A recent study demonstrated that inhibition of caspase-1 with tetracycline reduced IL-1β and IL-18 concentrations, lung injury, and outcome [49]. Another study showed that miRNA-495 attenuated LPS-induced lung injury by negatively regulating NLRP3 inflammasome-mediated pyroptosis in alveolar macrophages [50]. Shi et al. found that HSF1 protected ALI by inhibiting NLRP3 inflammasome and caspase1 activation [51]. In addition, increasing work has shown that caspase-11-mediated pyroptosis was involved in the development of ALI, and deletion of caspase-11 reduced lung injury [43, 52]. These above published findings have indicated that inhibition of canonical or non-canonical pyroptosis pathway could alleviate ALI. Additionally, it has been reported that cardiac-specific knockdown of *Bhlhe40* attenuated Ang II-induced activation of NLRP3 inflammasome pathway in the atria [53]. Nevertheless, the extract relationship between Bhlhe40 and pyroptosis pathways during ALI has not been reported. In most previous studies, researchers pay attention to investigate either canonical or non-canonical pyroptosis pathway, and rarely examined both pathways at the same time. Consequently, our study focused on the two pyroptosis pathways to study the pathogenesis of ALI. In line with pervious study, our data demonstrated that canonical and non-canonical pyroptosis pathways were both activated in LPS-induced mice and macrophages, whereas they were inhibited by *Bhlhe40* deficiency both in vivo and in vitro.

This study still has several limitations. Firstly, LPS-induced ALI and infection models differ fundamentally, although LPS is one of the first targets recognized by the host immune system during infections. And this may explain why previous studies found increased susceptibility of mice lacking Bhlhe40 during lung infection by Mycobacterium tuberculosis. Thus, it needs necessary to be studied further in different pathogens infection models. Secondly, while our study focused on the role of Bhlhe40 in macrophages, a function of Bhlhe40 on other cell types, including several non-immune cells, cannot be ruled out. Therefore, macrophage-specific *Bhlhe40* knockout mice are needed in future studies to further confirm our findings. Thirdly, Bhlhe40, as a transcription factor which acts on a specific sequence, did not explore its specific target genes. The transcription regulation mechanism of Bhlhe40 remains controversial, it would be necessary to investigate in the further. Finally, animal experiments were only conducted in adult male mice, and although highly unlikely, we cannot exclude the possibility that some of the responses of female mice could be different.

## Conclusion

In summary, our major finding in this study is the identification of Bhlhe40 as a key pro-inflammatory regulator in lung inflammation and injury. Mechanistically, *Bhlhe40* deficiency regulates caspase-1 and caspase-11 mediated inflammatory pathway inactivation and therefore attenuates GSDMD-mediated macrophage pyroptosis. Therefore, Bhlhe40 may be a potential therapeutic target for the treatment of ALI.

### Electronic supplementary material

Below is the link to the electronic supplementary material.

Supplementary Material 1

Supplementary Material 2

Supplementary Material 3

## Data Availability

All data generated or analyzed during this study are included in this published article [and its supplementary information files].
