# Supplementary material for: Bhlhe40 deficiency attenuates LPS-induced acute lung injury through preventing macrophage pyroptosis
Source: Respir Res. 2024 Feb 24;25:100. doi: 10.1186/s12931-024-02740-2 (PMC10894472; doi:10.1186/s12931-024-02740-2)

**Supplemental Figure 1** Disulfiram ameliorated lung inflammation in WT mice treated with LPS. (A) The mRNA expression of *Gsdma*, *Gsdmc*, *Gsdmd* and *Gsdme* in the lung of mice treated with LPS were assessed by qRT-PCR. n = 6. (B) WT mice were induced by LPS and then treated with different dose of disulfiram (25mg/kg, 50mg/kg and 100mg/kg). The mRNA expression of *Il-6*, *Tnf-a*, *Mcp-1* and *Cxcl10* in the lungs of mice were assessed by qRT-PCR. n = 3. Data are shown as the mean ± SEM. Statistical analysis was performed by one-way ANOVA followed by Bonferroni's multiple comparisons test or unpaired two-tailed Student’s t-test. ***p* < 0.01, ****p* < 0.001, *^ns^p* > 0.05.


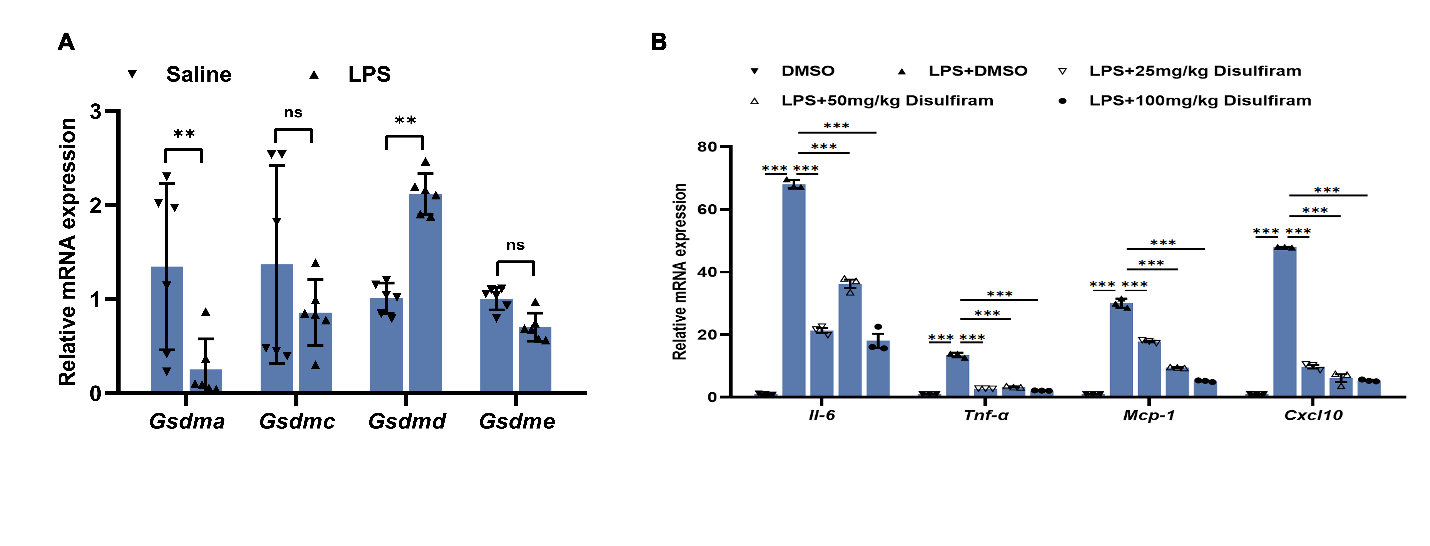

Supplement: Supplementary file 1 — Supplementary Material 1 [file 12931_2024_2740_MOESM1_ESM.docx]
