# Supplementary material for: Bhlhe40 deficiency attenuates LPS-induced acute lung injury through preventing macrophage pyroptosis
Source: Respir Res. 2024 Feb 24;25:100. doi: 10.1186/s12931-024-02740-2 (PMC10894472; doi:10.1186/s12931-024-02740-2)

**Supplemental Figure 2** *Bhlhe40* deficiency inhibits caspase-1-mediated and caspase-11-mediated inflammatory pathways in LPS-induced ALI mice. (A-B) Quantified analysis of GSDMD^NT^ and cleaved IL-1β by Western blot. (C) Quantified analysis of cleaved caspase-1, NLRP3 and ASC by Western blot. (D) The mRNA expression of *Casp1*, *Nlrp3,* *Asc* and *Casp11* in the lung of WT and *Bhlhe40^-/-^* mice were assessed by qRT-PCR. (E) Quantified analysis of cleaved caspase-11 by Western blot. n = 6. Data are shown as the mean ± SEM. Statistical analysis was performed by two-way ANOVA followed by Bonferroni's multiple comparisons test. **p* < 0.05, ***p* < 0.01, ****p* < 0.001.


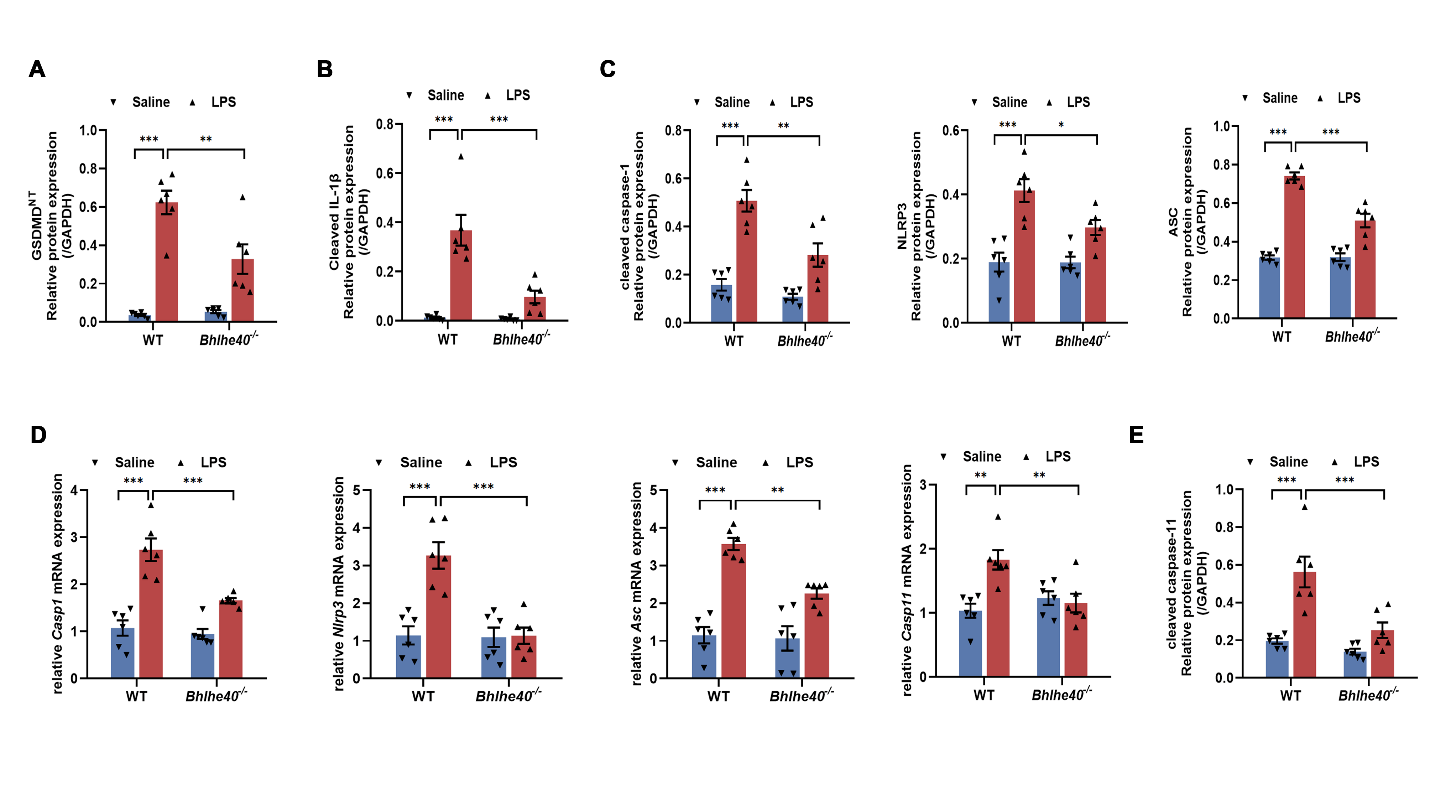

Supplement: Supplementary file 2 — Supplementary Material 2 [file 12931_2024_2740_MOESM2_ESM.docx]
