# Supplementary material for: Bhlhe40 deficiency attenuates LPS-induced acute lung injury through preventing macrophage pyroptosis
Source: Respir Res. 2024 Feb 24;25:100. doi: 10.1186/s12931-024-02740-2 (PMC10894472; doi:10.1186/s12931-024-02740-2)

**Supplemental Figure 3** Bhlhe40 was highly expressed in LPS-stimulated macrophages. (A-B) The protein level of Bhlhe40 in BMDMs were detected by western blots and quantified analysis. (C-D) Quantified analysis of GSDMD^NT^ and cleaved IL-1β by Western blot. (E) Quantified analysis of cleaved caspase-1, cleaved caspase-11, NLRP3 and ASC by Western blot. n = 3. Data are shown as the mean ± SEM. Statistical analysis was performed by two-way ANOVA followed by Bonferroni's multiple comparisons test or unpaired two-tailed Student’s t-test. **p* < 0.05, ***p* < 0.01, ****p* < 0.001.


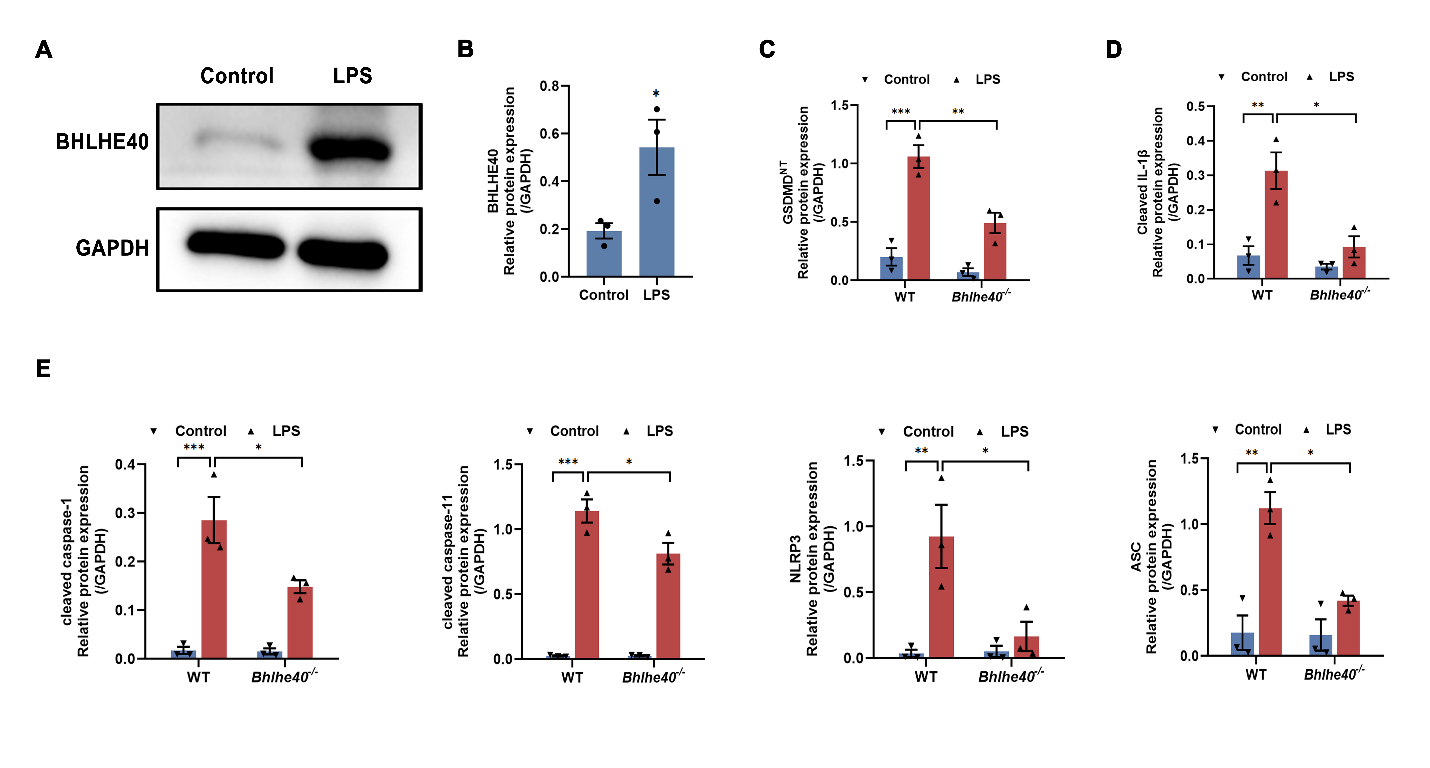

Supplement: Supplementary file 3 — Supplementary Material 3 [file 12931_2024_2740_MOESM3_ESM.docx]
